# Pregnant Women’s perceptions of exposure to brominated flame retardants

**DOI:** 10.1186/s12978-016-0257-2

**Published:** 2016-12-01

**Authors:** A. Lane, C. G. Goodyer, F. Rab, J. M. Ashley, S. Sharma, A. Hodgson, J. Nisker

**Affiliations:** 1 Department of Obstetrics and Gynaecology, Schulich School of Medicine & Dentistry, The University of Western Ontario, London, Ontario Canada; 2 Department of Pediatrics, McGill University, Montreal, Quebec Canada; 3 Children’s Health Research Institute, London, Ontario Canada; 4 Obstetrics and Gynaecology, Schulich School of Medicine & Dentistry, Scientist, Children’s Health Research Institute, The University of Western Ontario, London Health Sciences Centre, Victoria Hospital, Rm E2-620E, 800 Commissioners Rd E., London, Ontario N6A 5W9 Canada

**Keywords:** Brominated flame retardants, Endocrine disruption, Household chemicals in pregnancy, Polybrominated diphenyl ethers (PBDEs), Maternal exposure, Prenatal exposure

## Abstract

**Background:**

Recent media reports on human studies associating brominated flame retardants (BFRs) in household products in pregnancy with urogenital anomalies in boys and endocrine disruption in both sexes. We sought to explore the perceptions of pregnant women of brominated flame retardant (BFR) exposure, in light of recent media reports on the adverse health effects of BFR exposure prenatally.

**Methods:**

Pregnant women were recruited for interviews through posters and pamphlets in prenatal clinics, prenatal fairs and community centres. Interviews were audiotaped and transcribed verbatim for Charmaz-based qualitative analysis supported by NVIVO 10™.

**Results:**

Theoretical sufficiency was reached after analyzing the interviews of 23 pregnant women. Themes co-constructed were: I–Lack of Awareness of BFRs; II–Factors Influencing BFR Exposure; III–Responsibility; IV–Informed Choice. Almost all participants felt it was difficult to make informed choices to avoid BFRs, and wanted communication from clinicians and regulation from governments regarding decreasing BFR exposure.

**Conclusion:**

Pregnant women in Canada may be unaware of the potential risks of exposure to BFRs. Professional organizations and governments should further study risk associated with BFR exposure in pregnancy and provide educational materials for pregnant women and clinicians regarding BFR exposure.

## Plain English summary

Increased media attention has reflected the research suggesting exposure to brominated flame retardants (BFRs) may be associated with harmful health effects in pregnancy. However, to date there has been no action taken by clinicians, professional organizations or governments to inform pregnant women of the widespread use and potentially harmful health effects of BFR exposure and the potential to reduce exposure. Our research was undertaken to explore the perceptions of pregnant women regarding BFR exposure in pregnancy. With Research Ethics Board approval, pregnant women from Southwestern Ontario Canada were recruited through the use of posters and pamphlets in prenatal clinics, prenatal fairs and community centres. The interviews were audiotaped and transcribed word for word. The transcripts of the interviews were analyzed using a rigorous research method called “qualitative analysis” including NVIVO 10™ software. After 22 pregnant women had been interviewed and their transcripts analyzed, it was felt no new information was being offered, and thus no additional pregnant women were recruited. Four themes were co-constructed: I-Lack of Awareness of BFRs, II-Factors Influencing BFR Exposure, III-Responsibility and IV-Informed Choice. Many participants felt that flame retardants were difficult to avoid through individual means. In the future, they wanted better communication from their clinicians and governments, and regulations that would promote decreased exposure. Pregnant women in Canada may be unaware of the potential risks of exposure to BFRs. Professional organizations and governments should further study risk associated with BFR exposure in pregnancy and provide educational materials for pregnant women and clinicians regarding BFR exposure.

## Background

Brominated flame retardants (BFRs) are added to household products such as furniture and textiles to decrease their flammability [1]. BFRs are released into the air as dust and are subsequently inhaled or consumed with food [2]. Contact with contaminated dust accounts for approximately 82% of human exposure [2]. BFRs cross the placenta [3] and their levels in human umbilical cord serum have been shown to be significantly higher than levels in maternal serum [4]. In addition, BFRs are found in breast milk [5]. These data suggest that the fetus and postnatal infant may be highly exposed to BFRs via their maternal environment.

Women are exposed to many endocrine disrupting chemicals at a low level [6, 7], including BFRs, throughout their life and during pregnancy. These exposures could potentially act additively or have a synergistic effect [8]. However, there remains considerable uncertainty about the risks of exposure to these complex chemical mixtures since scientific methods to adequately assess risk have not yet been developed [8].

Prenatal BFR exposure in humans has been associated with signs of endocrine disruption, including abnormal male genitourinary development (cryptorchidism) [9], hernia and hydrocele [10], and alterations in the thyroid hormone axis as well as male hormone levels [11, 12]. Animal studies also report that exposure to BFRs is associated with several indices of endocrine disruption [13, 14], including androgen signaling and thyroid changes [15], in addition to hepatotoxicity [16, 17] and abnormal neurobehavior [18].

Numerous studies have shown that some of the highest concentrations of flame retardants within our everyday environment occur in North America [19]. Biomonitoring studies in the United States and Canada indicate a ten to one-hundred fold higher serum levels of BFRs compared to populations in many other regions of the world [19]. Increased media attention on these BFR studies may be raising anxieties in North American pregnant women and women planning to become pregnant [8].

As clinicians providing obstetrical and preconceptual care may not be generally informed about BFRs except through the same media attention their patients may receive, the responsibility falls on professional organizations interested in reproductive health to provide comprehensive and accessible resources for clinicians as well as for pregnant women. In the United Kingdom, the Royal College of Obstetricians and Gynaecologists published a scientific impact paper entitled “Chemical Exposures During Pregnancy: Dealing with Potential, but Unproven, Risks to Child Health” to raise awareness of the current issues surrounding chemical exposure by issuing a list of products which pregnant women and women contemplating pregnancy should avoid to minimize harm to their pregnancy [8]. In addition, professional development tools such as “Environmental Impacts on Reproductive Health” by The Association of Reproductive Health Professionals (ARHP) [20] and an editorial entitled “Potential Toxicity of Synthetic Chemicals: What You Should Know About Endocrine-Disrupting Chemicals” in the American Family Physician are resources for clinicians [21]. The American Congress of Obstetricians and Gynecologists (ACOG) and the American Society for Reproductive Medicine (ASRM) in 2013 showed concern about endocrine disrupters, and have urged the Environmental Protection Agency (EPA) to take measures “to ensure the safety of all mothers and infants from toxic environmental agents [22]. However, neither ACOG nor ASRM provide specific guidance to clinicians as to how to discuss avoidance of endocrine disruptors in general or BFRs in particular.

There has been no action taken by Canadian clinicians, professional organizations or governments to inform pregnant women and women planning pregnancy of the widespread use and potentially harmful health effects of BFR exposure and the potential to reduce exposure. The purpose of this study was to explore pregnant women’s perceptions of exposure to BFRs and their views on the role of clinicians as well as the government in providing appropriate information about BFRs.

## Methods

### Recruitment

Pregnant women from Southwestern Ontario were recruited through posters and pamphlets in prenatal clinics, prenatal fairs and community centers.

### Interviews

Semi-structured 20 to 40 min interviews were conducted by three members of the research team (JA, SS, AH), which were audiotaped and transcribed verbatim. Non-leading interview prompts were used to generate conversation and capture individual perceptions, understandings and experiences at great length. While flame retardants were used as an example of an everyday household chemical, participants were encouraged to discuss any household chemicals. Pauses and notes on emotional tone of spoken text were also incorporated into the transcripts. All identifying information was removed from the transcripts and the audiotapes were erased to ensure confidentiality and anonymity and to protect the rights of the participants [23].

### Analysis

The data underwent rigorous qualitative analysis using constructionist grounded theory methodology [24] supported by NVivo™ 10 software (QRS International Proprietary Limited, Doncaster, Victoria, Australia). In the independent (AL, FR) initial coding phase, the data were broken down into smaller, salient codes using line- by-line coding [25], then the data were recoded based on the most significant or frequent codes to generate focused codes. The categories generated during the coding process were used to develop the emerging subthemes and themes and constant comparison was used to align and sharpen these themes [24, 25]. Theoretical sampling was conducted until no new themes emerged in the data. Theoretical saturation was reached after 23 pregnant women had been interviewed and their transcripts independently analyzed (AL, FR).

### Ethics

Research ethics approval was obtained from the Health Science Research Ethics Board (17406E) at the University of Western Ontario.

## Results

**Fig. 1:**
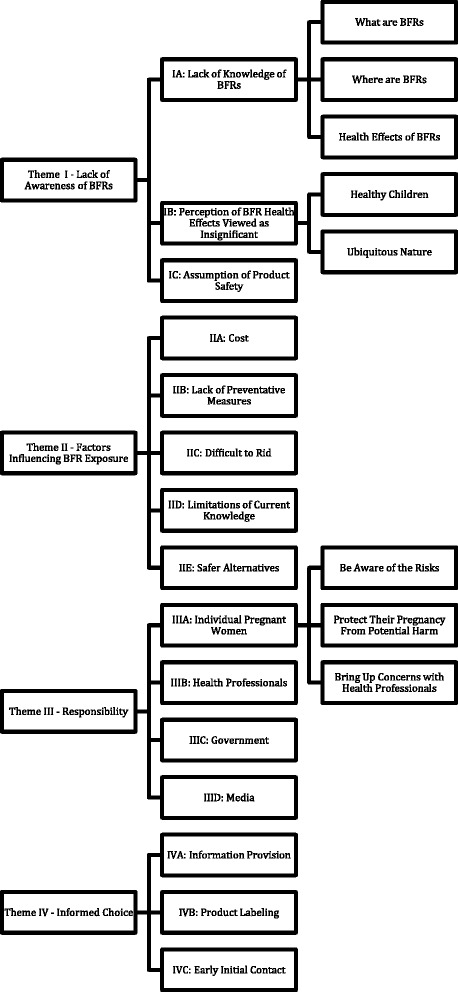
Overview of results of qualitative analysis

The results of the analysis have been organized under the four themes encompassing the concepts identified in the earlier phases of coding and reflect distinct issues and concerns: Theme I–Lack of Awareness of BFRs; Theme II–Factors Influencing BFR Exposure; Theme III–Responsibility; and Theme IV–Informed Choice. Figure 1 presents an overview of how the research participants’ comments were organized into the various categories and were then grouped into various subthemes and then further organized into these overarching themes. Sample excerpts from the transcripts illustrating each of the four themes are presented in Tables 1, 2, 3 and 4.

**Table 1:** Lack of Awareness of BFRs

THEME I: Lack of Awareness of BFRs	Comments
IA: Lack of Knowledge of BFRs	*“…flame retardants…I would probably like to know more about…exactly what product they are in…”* RP7 *“…how do you know what they spray on your stuff? Do they* [spray it on]*…everybody’s stuff?”* RP13 *“I haven’t heard why they’re dangerous, but I have heard that brominated stuff was in…car seats and stuff and that it’s obviously a chemical that’s inhaled…”* RP4 *“…I just heard about…something going up in flames, it’s best to get it sprayed…but I didn’t hear about the side effects or the concerns…”* RP5 *“…I think, it’s [in] insulation, in different…foams and things like that, but I didn’t know it would have effect on pregnancy, I’ve heard of it before, but mostly for its fire protectant attributes.”* RP12 *“…weren’t they using them on mattresses and pillows at one point and they pulled them off them…I do remember hearing that…if they pulled other things already then obviously…there’s something going on there…”* RP1 *“I see pajamas with flame retardant on it…I don’t really know what that means…so I never buy that, I always look for just cotton without any chemicals.”* RP11 *“I’m sure they served a purpose at one point, but why does my newborn need to have a flame retardant sleeper on her?”* RP9
IB: Perception of BFR Health Effects Viewed as Insignificant	*“…it’s everywhere…everyone’s been exposed to it…I suppose if you knew for sure, someone close to you, had that problem then I guess it would kind of hit home a little bit more.”* RP12 *“…a lot of these things* [such as BFRs]*…have been developed and people have got healthy children.”* RP3 *“…considering I have three beautiful, healthy children, no, not really* [concerned]*.”* RP9 *“…maybe, if I couldn’t get pregnant, I’d think…maybe I couldn’t get pregnant because of the stuff they spray on things* [BFRs]*…but, I don’t know.”* RP13
IC: Assumption of Product Safety	*“… if there’s a product in the shelf in Canada I would think it’s safe to use.”* RP21 *“…I shouldn’t have to take…everything I buy and say, is this safe… maybe I should question more but I don’t. I just take it* [product safety] *for granted…”* RP21 *“…I just assume if it’s for babies* [such as pajamas] *that it’s supposed to be safe for them…”* RP13 *“…I should be more concerned than I am…I don’t go out of my way to make sure that I’m not using…products with those chemicals* [such as BFRs]*, but then…how often to you see it labeled…”* RP9

**Table 2:** Factors Influencing BFR Exposure

THEME II: Factors Influencing BFR Exposure	Comments
IIA: Cost	*“You can only redo your home so much when you're having a baby, eventually finances get* [in the way]*…there's only so much you can do…for yourself and for your baby…”* RP1 *“Some stuff* [such as a couch] *is just a little too expensive to replace.”* RP20 *“…if…the stuff I use for cleaning…on the back…said don’t use…I would go out and buy the safe stuff…but, my couch…I’d have to know for sure… because that would cost a lot of money and it could be really unnecessary…”* RP22
IIB: Lack of Preventative Measures	*“…just about everything you buy you’re not going to get that information…at least what was used to make it…”* RP12 *“…if it’s* [BFRs] *not banned and every house has it in them how do you go around* [avoid it]*…”* RP15
IIC: Difficult to Rid	*“It seems unrealistic to get rid of all of these…wiring, insulation, plastics, textiles, and phones…it’s all over the place…How are you going to control that?”* RP18 *“TVs, can’t avoid them, computers, can’t avoid them, can’t avoid really any of those, carpets, furniture, kitchen appliances…they’re there.”* RP20 *“Well I think…it would impact anybody that reads that stuff* [potential harms of BFRs] *but how do you avoid it…it’s around us all the time…”* RP3 *“…we’d have to go through everything and see what’s where. I wouldn’t even know where to begin…”* RP15 *“…if it’s used everywhere it’s probably in this room in several forms…It’s hard to avoid unless you replace it as you go, as you upgrade things.”* RP12 *“…just about everything could be of concern…and we don’t know, I guess, kind of hard to avoid everything…especially it’s in your…daily living…the exposures, so kinda hard…”* RP5
IID: Limitations of Current Knowledge	*“It* [the harm from BFR exposure] *would need to be their* [the researchers] *concern, definitely a problem then for me to do something about it.”* RP6 *“…pretty significant. I would need to know whether it’s* [BFRs] *really harmful* [to change lifestyle]*…”* RP3 *“Well he* [the healthcare professional] *should be fairly certain that there’s a fairly high risk, but even if it’s* [BFRs] *a lower risk he should mention it if he knows you’re exposed to it.”* RP20 *“If they’re not sure then I don’t want to know* [about the potential risks of BFRs]*. Especially if like every day, like TVs, computers…I’m in front of the computer every day, all day; kitchen appliances, well I need to cook…and carpets…even if they were sure, how would you, how do you avoid that?”* RP18 *“…I would say only if you know that it’s really going to hurt people* [BFRs]*, I would think yes it should be a warning, but, you don’t want to alarm a whole bunch of people if you’re not really sure yet.”* RP3
IIE: Safer Alternatives	*“…they* [the government] *would have to come up with some sort of alternative…that is safe…”* RP8 *“They* [the government] *should be funding the research for alternatives. I can’t say that they should be doing the research themselves, or making them themselves, but, funding for the researchers to do it.”* RP10 *“…smoke detectors…keeping matches away from small children…there are other alternatives to having all the flame retardants sprayed on.”* RP7

**Table 3:** Responsibility

THEME III: Responsibility	Comments
IIIA: Individual Pregnant Women	*“…if they’re* [BFRs] *not labeled and I’m going to be exposed to them I could do some more research on my own to find out how it’s actually leaching out, what’s causing it…if I educated myself then I’d be able to…stay away from the things…”* RP9 *“Educating myself…on them* [the risks of BFRs].” RP8 *“You yourself are responsib*[le] *for yourself and your unborn child…”* RP5 *“…it’s on you to…protect your child, or to make… choices for your child.”* RP19 *“…it’s parents’ responsibility to try…to look out for their children and do the best they can to keep them healthy, particularly in the womb when they’re most vulnerable.”* RP11 *“…I just feel like I want to be in a bubble around them* [my children]*, so I can protect them.”* RP22 *“…not unless I was the first to bring it up…cause… he* [the health professional] *may not know if they’re exposed to it.”* RP20 *“…I think the responsibility does come on the women to bring up her…concern and try and find other resources…where they can find more information.”* RP19 *“…I hope the doctor would bring it* [BFRs] *up but if I hear something that the doctor hasn’t said then I usually bring it up to him.”* RP19
IIIB: Health Professionals	*“…if they* [health professionals] *could only give more information* [on BFRs] *then we can make wiser decisions but not always leaving it up to the person to go find that information because sometimes they don’t even know, like, it’s a problem in the first place…”* RP16 *“I would believe that the healthcare provider could help give an insight to it…and unfortunately that’s the only person that’s going to be able to do it…”* RP9 *“I think your health professionals, definitely, should be relaying that information* [of BFRs] *to patients.”* RP7 *“…I think that the doctor should tell you about most of the things* [such as the potential risks of BFRs] *cause, like, I mean it’s sort of, you’re going to the appointment to because you’re pregnant.”* RP2
IIIC: Government	*“…if it was the government that regulated that it had to be on it* [household products] *then it should be the government’s responsibility telling everyone what it actually is.”* RP9 *“…the government…they should have regulations, they should be banning this stuff* [BFRs]*.”* RP11 *“…Minister of Health and Longterm Care should have some impact about putting the stuff* [information on the potential risks of BFRs] *out there…”* RP1 *“…there should be studies going on, and government giving money to studies to make sure we can reduce the risk of any problems down the road…”* RP7 *“The researchers are going to come up with it* [risks of BFRs]*…but they don’t have the money to do the advertising…so…the researchers* [should] *first tell the government so the government should tell us…”* RP9
IIID: Media	*“*[The media] *brings it to the attention of a lot more people…so I think they would influence a lot of people.”* RP12 *“…I think the media should talk about this* [BFRs]*…”* RP14 *“…I think media should, would be a good option because by then you’re already into your pregnancy and I think it would be important to know about this* [BFRs] *beforehand.”* RP7

**Table 4:** Informed Choice

THEME IV: Informed Choice	Comments
IVA: Information Provision	*“…there’s…so many hazards for the mother and the baby that just…providing a good source of information* [on BFRs] *would be helpful.”* RP12 *“…I guess it should be known that there is a concern in regards to the chemical that is being used* [such as BFRs] *and leave it up to the individual…let them…be known it’s a risk…”* RP5 *“…if the person has the choice, if they go on the government resource…you could at least look at that and see, well, they’re saying* [BFRs]*…could be risk, so I don’t need to* [be concerned] *but I could…”* RP4 *“…I think there should be…a warning link…if there is even a hint of possible threat to our lives…it should definitely be brought to everyone’s attention and then it’s for people to choose whether they want to go from there.”* RP3 *“…pamphlets at doctors’ offices would probably work the best.”* RP3 *“…I think that we should have the opportunity to know everything that you would, say, a drug that a doctor’s prescribing you…or a procedure that you’re going to have done, you should be able to make the informed decision on your own instead of having it* [BFR exposure] *forced upon you.”* RP9
IVB: Product Labelling	*“…I don’t think I’d go as far as prohibiting their* [BFRs] *use. I would say the labeling, letting the consumers make a choice…you can’t just say, oh but there’s nothing wrong until we find out…where consumers are aware, things are clearly labeled…the information’s out there for people to get to make the choices that they need.”* RP9 *“…a warning label would be something that would be considered. I would always look for that cause I do generally read labels, so, something visual on the product would really stand out and makes you think twice about purchasing it* [products containing BFRs]*.”* RP7 *“…it’s* [products containing BFRs] *no different than the genetically modified food, they should be labeled as well, so that we can make our own informed choices…”* RP9
IVC: Early Initial Contact	*“…there’s a* [BFR] *risk to pregnant women…you should be informed before you get pregnant so that you can avoid those risks…”* RP4 *“I would want to hear about it before I was pregnant, if I had the chance. Cause I would want to avoid it through the whole pregnancy if possible.”* RP19 *“I think it* [discussion about the potential harms BFRs] *would have to be done preventatively… probably through a healthcare professional if you’re within childbearing age your physician will recommend folic acid, or making sure you’re taking your supplements. So they probably should be knowledgeable about this so they can also talk to you about this preventatively…”* RP7

### Theme I–lack of awareness of BFRs

The first theme was a lack of awareness of BFRs and their potential health effects. Sample excerpts from some of the comments that led to Theme I are illustrated in Table 1. The subthemes that were grouped into Theme I were: IA) Lack of Knowledge of BFRs; IB) Perception of BFR Health Effects; and IC) Assumption of Product Safety.

Most of the research participants indicated no awareness of flame retardants, and the knowledge of the few who showed some level of awareness related only to its attributes as a fire protection agent rather than any potential harmful health effects. Several of the research participants commented that since they had not been warned about, heard about or personally seen health effects related to BFR exposure, they were unlikely to perceive the risk of BFR exposure as significant. For example, those participants who had given birth to healthy children in previous pregnancies were not as concerned about being exposed in subsequent pregnancies. There was a general assumption among the research participants that the products they were purchasing must be safe to use or the government would have banned them and manufacturers would not be able to use them.

### Theme II–factors influencing BFR exposure

The second theme encompassed factors relating to BFR exposure and avoidance. Excerpts from comments that relate to Theme II–Factors Influencing BFR Exposure are presented in Table 2. Five subthemes led to this theme: IIA) Cost; IIB) Lack of Preventative Measures; IIC) Difficult to Rid; IID) Limitations of Current Knowledge; and IIE) Safer Alternatives.

Many of the research participants identified cost as a limiting factor in their ability to reduce exposure to BFRs in the future. Several of the research participants contrasted reducing BFR exposure with reducing exposure to other household chemicals that were less costly to avoid or do not require major purchase (such as new furniture) to reduce exposure. Another factor that the research participants considered was the lack of preventive measures to assist in managing their exposure. They felt that the appropriate safety nets were not in place, such as product labeling and governments banning their use. Another factor that the research participants considered was the limited scientific research of the potential harm of exposure to flame retardants. A majority of the research participants reported that, until the risk is shown to be significant, they would not consider modifying their level of exposure. While some of the participants discussed the need for the government to develop safer alternatives, others suggested that fire protection could be achieved through safer means without the use of flame retardants. Overall, the research participants perceived flame retardants as difficult to personally avoid.

### Theme III–responsibility

The research participants felt that the responsibility to inform individuals about the potential harms of flame retardants may lie within several areas. Four subthemes were collected under Theme III–Responsibility: IIIA) Individual Pregnant Women; IIIB) Health Professionals; IIIC) Government; and IIID) Media. Sample excerpts from some of the comments that led to Theme III are illustrated in Table 3.

The research participants felt responsible to become aware of the potential risks of flame retardants and other substances during pregnancy to protect their pregnancy from potential harms. The research participants also felt responsible to bring their concerns about flame retardant exposure to their health professionals. Some felt that health professionals were not always reliable sources regarding this type of information as illustrated in their comments suggesting that physicians may lack time or awareness, and others that felt this type of information may be beyond the scope of medical care. However, the research participants wanted to receive information about the potential risks of flame retardants from their health professionals, as they viewed health professionals to be more trustworthy than any other source of information. Although the research participants showed a general distrust for the government, many felt that it was the government’s responsibility to educate individuals on the risk of flame retardants as well as cover the costs of future research to generate a better understand of the potential harms of flame retardants. Lastly, although there was some skepticism of the media, most of the research participants felt that the media was an effective tool to bring attention to the risks of flame retardants.

### Theme IV–informed choice

A theme relating to informed choice was identified. Excerpts from some of the comments made by the research participants regarding information they required to make informed decisions for themselves regarding their exposure to flame retardants are presented in Table 4 organized under the subthemes: IVA) Information Provision; IVB) Product Labeling; and IVC) Early Initial Contact.

The research participants discussed the need for resources, such as a website or pamphlets, to which individuals could be directed in order to acquire information on flame retardants. They also felt product labeling was essential for informed choice. In addition, the research participants wanted to receive this information prior to pregnancy, so that they could act in a preventative fashion. Overall, until research is able to clarify the precise health risks of BFR exposure with greater certainty, the research participants emphasized the importance of being informed about the potential risks of BFR exposure so that they could make their own choices.

## Discussion

In this study, pregnant women consistently believed that there is a responsibility of clinicians and governments to inform women about BFR exposure in pregnancy and strategies for avoidance, as long as Canadians continue to be exposed to BFRs. Increasing awareness of the potential harms of flame retardant exposure in pregnancy allows the individual woman to be aware of exposure to BFRs and to make informed decisions to reduce exposure to BFRs if possible [8]. However, lack of information sources on routes of exposure and potential harms of flame retardants in consumer products makes it difficult for pregnant women and women contemplating pregnancy to make such informed choices regarding how and to what extent they may decrease their exposure to flame retardants [26, 27].

Investigative media reporting may bring awareness of the risks of BFR exposure in pregnancy to these women, but is not a sufficient information source for informed choice [27].

Further study is needed to determine the risk associated with BFR exposure during pregnancy. At the current time, scientific methods to adequately assess risk of exposure to BFRs has not been developed [8]. A significant limitation in developing adequate methodologies has been the ubiquitous nature of BFRs preventing comparison between exposed and unexposed groups given that no unexposed groups exist. As such, future methods may benefit from dose response analysis to determine if there is a threshold exposure limit.

As Canadian clinical practice guidelines on BFRs do not currently exist, it is difficult for clinicians caring for pregnant women and women planning pregnancy to discuss flame retardant exposure. Without the possibility of such discussion with clinicians and lack of reliable public information strategies, such as by Health Canada, informed choice regarding avoidance of BFRs does not exist. Regulations can play an important role in shaping what is viewed as healthy behaviors during pregnancy and the “health of the embryo” [28], however, regulations on the use of BFRs and the impact of BFR-containing products do not currently inform Canadians of household exposure [29]. Furthermore, even if stringent regulations were indeed implemented, individuals will likely continue to be exposed by previously manufactured products treated with BFRs, which could remain in daily use for decades [30, 31].

Zota et al [32] found that significantly higher levels of flame retardants have been measured in individuals of low socio-economic status. This problem is likely due to non-toxic alternatives being generally more expensive than mainstream brands and requiring available time to shop for chemical-free products. Consequently, mothers with higher incomes may be able to shop their way around BFR exposure, while mothers with low incomes may have a limited capacity to carry out the safest choices [26]. Additionally, low income families may be more likely to have old furniture with exposed foam. In order to provide universal protection for the public, it is necessary for precaution to be enacted at the regulatory level, rather than at the level of the individual consumer [26, 27].

As pregnant women in the United States and Canada are exposed to much higher concentrations of BFRs than many other countries in the world [19], we encourage the American Congress of Obstetricians and Gynecologists, the Society of Obstetricians and Gynaecologists of Canada, and other national organizations of physicians and midwives, and governments to further study risk associated with BFR exposure in pregnancy, as well as implement public education strategies to raise awareness and provide potential avoidance strategies for BFR exposure during pregnancy, and to provide educational materials for clinicians to discuss BFR exposure with their patients.

## Conclusion

The research participants consistently felt that as long as women continue to be exposed to BFRs there is a responsibility of clinicians and governments to inform pregnant women and women contemplating pregnancy about exposure to BFRs and strategies for avoidance. The American Congress of Obstetricians and Gynecologists, other national organizations of physicians and midwives, and governments should further study risk associated with BFR exposure in pregnancy, as well as provide public education strategies to raise awareness and provide potential avoidance strategies, and educational materials for clinicians to discuss flame retardant exposure with their patients.

## Abbreviations

ACOG: American Congress of Obstetricians and Gynecologists
ARHP: The Association of Reproductive Health Professionals
ASRM: American Society for Reproductive Medicine
BFR: Brominated flame retardant
PBDEs: Polybrominated diphenyl ethers
